# Longer Wait Times Do Not Adversely Impact 90-Day Mortality in Patients With Stages I-III Gastric Cancer

**DOI:** 10.7759/cureus.46494

**Published:** 2023-10-04

**Authors:** Siddharth Ramanathan, Nathan Shen, Thomas Johnson, Chin Cheng, Faiz Tuma, Eduardo Serpa, Maher Ghanem

**Affiliations:** 1 Medicine, Oakland University William Beaumont School of Medicine, Rochester Hills, USA; 2 Hematology and Oncology, Central Michigan University College of Medicine, Mount Pleasant, USA; 3 Oncology, Central Michigan University College of Medicine, Mount Pleasant, USA; 4 Statistics, Central Michigan University College of Medicine, Mount Pleasant, USA; 5 Surgery, Central Michigan University College of Medicine, Mount Pleasant, USA; 6 General Surgery, Central Michigan University College of Medicine, Mount Pleasant, USA

**Keywords:** mortality, treatment delay, wait time, stage i-iii, gastric cancer

## Abstract

Introduction

Gastric cancer is one of the leading causes of cancer-related death in the United States. Surgery remains integral to the curative management of non-metastatic gastric cancer. However, delays to the date of surgery for gastric cancer patients are commonplace. To investigate the impact of treatment delays on gastric cancer mortality, we conducted a multivariable analysis of over 36,000 patients.

Materials & methods

After querying the National Cancer Database and excluding patients who did not meet inclusion criteria, our sample included 36,598 patients with stage I-III gastric cancer. We ran multivariable logistic regressions by regressing 90-day mortality on wait time. Other co-variables included sex, race, age, area of residence, comorbidities, insurance, histology, tumor grade, tumor stage, resection margins, treatment facility type, and treatment with chemotherapy.

Results

Our results demonstrated that each day of increased waiting time is associated with a 0.5% decrease in 90-day mortality. Other statistically significant predictors of higher 90-day mortality risk included male sex, black or white race, living in a small metropolitan or non-metropolitan area, older age, more severe comorbidities, non-private insurance, non-gastric stromal tumor cancer, non-well differentiated tumors, worse clinical stage, residual cancer, treatment at non-academic center, and no adjuvant/neoadjuvant chemotherapy.

Conclusion

These findings demonstrate that patients with longer wait times until surgery do not experience worse outcomes. These results are reassuring and can be cited to alleviate patient concerns.

## Introduction

Gastric cancer is the 5th most common neoplasm, and 3rd deadliest cancer worldwide [1]. Although gastric cancer mortality has been declining in most areas of the world, gastric cancers continue to portend poor prognoses as they are often asymptomatic malignancies that present at an advanced stage in the disease course [2]. The most prevalent of gastric cancer - gastric adenocarcinoma - constitutes 95% of all gastric cancers, and can be further divided into a cardia and non-cardia subtype. While innovations in the treatment of Helicobacter pylori and gastroesophageal reflux disease (GERD) have decreased the overall incidence of non-cardia gastric adenocarcinoma; they have simultaneously contributed to a seven-fold increase in cardia gastric cancers [3]. As such, it becomes necessary to critically evaluate factors that may contribute to mortality in patients diagnosed with gastric cancer.

In the United States, the survival rates for gastric carcinoma are among the worst of any solid tumor. To date, surgery, with or without adjuvant/neoadjuvant chemotherapy remains the only curative treatment. Adjunct treatments with neoadjuvant/adjuvant chemotherapy or chemoradiation are utilized to improve outcomes following surgical resection [4]. Screening and diagnostic measures have also contributed to the reduction in gastric cancer mortality. In Japan, the introduction of early gastric cancer screening protocols using double contrast radiography and endoscopy has resulted in dramatic improvements in five-year survival [5]. Early detection of gastric cancers has allowed for effective local treatment, and thereby reduced overall healthcare burden [6]. While these protocols have certainly improved the overall survival, there is no clear consensus regarding the factors underpinning poor surgical outcomes in patients with gastric cancer.

The association between delays to treatment and post-treatment outcomes has been investigated in various paradigms across different countries and healthcare systems. Importantly, waiting time and treatment outcomes have differing definitions in the literature, adding nuance to the interpretation and generalizability of these findings. Nonetheless, these results are vital in informing policy and clinical recommendations for patients diagnosed with gastric cancer.

This study examines the relationship between time to treatment and gastric cancer survival across a large sample of patients in the United States treated between 2004-2018. In this analysis, we evaluate demographic, social, and treatment factors that may potentially impact the association between delays to treatment and overall survival in gastric cancer patients using one of the largest published datasets. Compared to previously published studies, this investigation analyzes a larger sample of patients and controls for variables not previously discussed to assess the impact of delays to treatment on patient outcomes in the United States. In this multivariable regression analysis, the primary outcome variable is the relationship between delays to treatment and 90-day mortality. Secondary outcome measures encompass the relationship between other covariates and 90-day mortality.

## Materials and methods

Multivariable regression model

$\Phi i = \frac{e^{\beta\tiny0 \space\normalsize +\space \normalsize\beta\tiny1\normalsize X\tiny1i \space\normalsize +\space \normalsize\beta\tiny2\normalsize X\tiny2i \space\normalsize +\space ... \space\normalsize +\space \beta\tiny13\normalsize X\tiny13i}}{1\space\normalsize +\space e^{\beta\tiny0 \space\normalsize +\space \normalsize\beta\tiny1\normalsize X\tiny1i \space\normalsize +\space \normalsize\beta\tiny2\normalsize X\tiny2i \space\normalsize +\space ... \space\normalsize +\space \beta\tiny13\normalsize X\tiny13i} }$

This study employs multivariable logistic regression to assess the effect of clinical variables on 90-day mortality in patients who underwent surgery for gastric adenocarcinoma. Our model is depicted above where Yi represents whether patients died 90 or fewer days after undergoing surgery, X1, . . ., X12 and X13 represent the design matrices for the variables age, sex, race, area of residence, Charlson-Deyo Comorbidity Index score, insurance status, histology, tumor differentiation, clinical stage, surgical margins, treatment facility, chemotherapy, time to treatment. This is a multivariable logistic regression where the parameters, βo, . . . , β14, are estimated by the maximum likelihood method.

Data source

Adult patients with non-metastatic gastric cancer were queried from the National Cancer Database (NCDB). These data represent over 70% of newly diagnosed malignancies in the United States [7]. Due to the nature of this study being a retrospective review of a deidentified, publicly available dataset, IRB or ethics committee approval was not required.

Exclusion criteria

The initial query of the NCDB resulted in 45,360 unique patients treated between 2004 and 2018. From this set, we excluded patients who received non-chemotherapeutic treatments (hormone therapy, endocrine therapy, or immunotherapy). Patients were also excluded if the use of chemotherapy was classified as indeterminate or unknown. From the remaining 43,665 patients, we excluded a further 7,067 records with incomplete data. The remaining 36,598 were analyzed as described below.

Data categorization

Data on age at diagnosis, sex, race, area of residence, Charlson-Deyo score, insurance status, histology, tumor differentiation, clinical stage, surgical margins, treatment facility classification, chemotherapy, and days between diagnosis and treatment were collected.

Race

The NCD contains a total of 31 racial classifications. The data show that the majority of patients fall into the “Black” (race code 1) and “White” (race code 2) classifications (90.7%). As such, the remaining race categories (race codes 3-99) were grouped as “Other”.

Area of Residence

The United States Department of Agriculture (USDA) Economic Research Service designates regions on an urban-to-rural spectrum using the population and proximity to metropolitan areas. The NCD variable UR_CD_03 was utilized to categorize patient residence at the time of diagnosis according to the definitions published by the USDA. Residential data were reclassified into three major categories: “Large metropolitan areas” with a population > 1 million (urban/rural code 1), “Metropolitan areas” with a population between 250,000 - 1 million (urban/rural code 2), and “Non-metropolitan areas” (urban/rural code 3-9).

Primary Payor at Diagnosis

Insurance data from NCD were obtained from patient questionnaires and existing records according to the North American Association of Central Cancer Registries (NAACCR) criterion. In our model, insurance status was classified as “Medicare” (NAACCR 630 codes 60-64), “Medicaid” (NAACCR 630 codes 31, 35), “Private insurance” (NAACCR codes 10, 20, 21), and “Other” (NAACCR 630 codes 1, 2, 65-68, 99).

Histology

In the NCD, histology was categorized according to ICD-0-3 guidelines. We reclassified histological data into four major groups: “Gastric adenocarcinoma” (ICD-0-3 code 8140, 8144), “gastrointestinal stromal tumor” (GIST) (ICD-0-3 code 8936), “Signet Ring Carcinoma” (ICD-0-3 code 8490), and ”Other” (all remaining ICD-0-3 codes).

Grade

Tumor grade was also classified according to ICD-0-3 guidelines, and coded as in the NCD. We regrouped tumor grades into four categories: “Well Differentiated” (NCD code 1), “Moderately Differentiated” (NCD code 2), “Poorly Differentiated” (NCD code 3), and “Other” (NCD codes 4-9).

Stage

Tumor stage was classified according to criteria specified in the American Joint Committee on Cancer (AJCC) 7th edition. No reclassifications were done for this analysis.

Surgical Margins

Surgical margins were categorized based on gross and microscopic findings from pathology reports. For this study, we categorized surgical margins as: “No Residual” (NCD code 0), “Residual” (NCD codes 1-3), and “Other” (NCD codes 7-9).

Treatment Facility

Facility type was reported according to guidelines set by the Commission on Cancer Accreditation program [8]. We reclassified Facility Type into “Community Center” (NCD codes 1-2), “Academic/Research Center” (NCD code 3), and “Integrated Cancer Center” (NCD code 4) [8].

Chemotherapy

The utilization of chemotherapy was classified based on when chemotherapy was administered relative to the date of surgery. This resulted in five categories: “No chemotherapy” (NCD code 0), “Chemotherapy before surgery” (NCD code 2), “Chemotherapy after surgery” (NCD code 3), “Chemotherapy before and after surgery” (NCD code 4), and “Other” (NCD codes 6-9).

Time Between Diagnosis and Treatment

The time between diagnosis and treatment was presented as a continuous variable in the NCD database and used without reclassification in our analysis.

Statistical analysis

Statistical analysis was primarily conducted using SAS software, version 9.4 (SAS Institute Inc., Cary, NC). Descriptive statistics are provided as count and percentage for categorical variables and as mean and standard deviation for continuous variables. Multivariable logistic regression modeling was used to identify variables associated with mortality within 90 days of the procedure. The analytical results were considered to be significant when the 95% confidence interval (CI) for odds ratio (OR) did not contain 1.

## Results

Demographic variables

Age

Accounting for all other covariates, patient age at the time of diagnosis (mean 65+/-12) was positively correlated to the odds of mortality within 90 days of surgery such that each additional year conferred a 4% increase in mortality rate (95% CI 1.03-1.05).

Gender

From the sample of 36,598 patients with non-metastatic gastric cancer, 65.2% (23,862) were male and 34.8% (12,736) were female. The analysis showed that male patients had 34% higher odds of dying within 90 days of surgery than female patients (95% CI 1.20-1.47).

Race

White participants made up 76.82% (28,114) of the sample, Black participants were 14.31% (5,237), and all other racial categories, including Asian, Pacific Islander, Alaskan native or any other category, composed the remaining 8.87% (3,247) of patients. When compared to white patients, our model showed that black participants were not at an increased mortality risk (95% CI 0.98-1.30), and that patients belonging to any other racial category had a 33% reduction in their risk of death within 90 days of surgery (95% CI 0.57-0.84).

Area of Residence

From our sample, 20,647 (56.49%) patients lived in large metropolitan areas, 10,769 (29.43%) patients lived in small metropolitan areas, and the remaining 5,155 (14.09%) patients lived in non-metropolitan areas. Compared to patients living in large metropolitan areas, our model showed that the odds of mortality within 90 days of surgery increased by 14% (95% CI 1.02-1.29) for patients residing in small metropolitan and increased by 28% (95% CI 1.12-1.45) for patients who lived in non-metropolitan areas.

Comorbidities and Social Determinants

To account for patient comorbidities, we utilized the Charlson-Deyo Comorbidity Index (CCI) score to provide a robust single-value estimate of non-tumor disease burden. In this analysis, 24,398 (66.66%) patients had a CCI score of 0 at the time of diagnosis, 8,510 (23.25%) patients had a score of 1, 2,517 (6.88%) patients had a score of 2, and 1,173 (3.21%) patients had a score of 3 or greater. This model demonstrated a 15% increase in the odds of mortality within 90 days of surgery for patients with a score of 1 as compared to 0 (95% CI 1.04-1.29), a 32% increase in odds for patients with a score of 2 compared to 0 (95% CI 1.12-1.54), and a 60% increase in odds for patients with a score of 3 or greater as compared to 0 (95% CI 1.26-1.94).

Insurance

The primary payor at the time of diagnosis was also assessed; 2,354 (6.43%) patients had Medicaid, 18,869 (51.56%) had Medicare, 13,932 (38.07%) had private insurance, and the remaining 1,443 (3.94%) had a different form of government insurance or no insurance (“Other Insurance Status”). This analysis demonstrated no difference in 90-day mortality from surgery between patients who had either Medicaid or Other Insurance Status as compared to Medicare. Patients insured with private insurance experienced a 14% reduction in the odds of mortality within 90 days of surgery compared to patients with Medicare (95% CI 0.76-0.98).

Tumor characteristics

Histology

Intestinal type adenocarcinoma comprised 26,125 (57.59%) patients, gastrointestinal stromal tumors (GIST) comprised an additional 5,816 (12.82%) patients, signet-ring cell histology comprised a further 6,696 (14.82%) patients, and the remaining 6,723 (14.78%) patients had malignancies of other histologic categorizations. The model identified no difference in the odds of mortality within 90 days of surgery for patients with signet-ring cell or other histologic types as compared to patients with intestinal-type adenocarcinoma; however, patients with GIST had a 76% reduction in the odds of mortality within 90 days of surgery as compared to those with intestinal-type adenocarcinoma (95% CI 0.18-0.31).

Grade

Regarding tumor differentiation, 4,097 (11.19%) patients had well-differentiated tumors, 9,949 (27.18%) patients had moderately-differentiated tumors, 17,941 (49.02%) patients had poorly-differentiated tumors, and 4,611 (12.60%) patients had other tumor grades. Compared to patients with well-differentiated cancers, this model demonstrated a 43% increase in the odds of mortality within 90 days of surgery for patients with moderately-differentiated tumors (95% CI 1.16-1.76), an 80% increase in odds of mortality for patients with poorly-differentiated tumors (95% CI 1.47-2.21), and a 32% increase in the odds of mortality for patients with other tumor grades (95% CI 1.03-1.69).

Clinical Stage

The AJCC 7th edition was used for disease staging - 15,444 (42.20%) patients were diagnosed with Stage I disease, 11,614 (31.73%) had Stage II disease, and 9,540 (26.07%) had Stage III disease. Compared to patients with stage I disease, patients diagnosed with Stage II disease experienced a 66% increase in their odds of 90-day mortality (95% CI 1.47-1.87) and patients with Stage III disease experienced a 107% increase in their odds of 90-day mortality (95% CI 1.82-2.36).

To ensure the validity of our results, we investigated whether there was a correlation between clinical stage and delays to treatment. The results demonstrated that patients with more severe disease based on clinical stage were more likely to be treated earlier than those with less severe disease. Average times to treatment were 40.0, 37.3 and 32.3 days respectively for patients with stage I, II, and III gastric cancer (p<0.001). To account for this correlation, we attempted to add an interaction term to include the combined effect of delays to treatment and clinical stage in our model. However, this variable was not a statistically significant predictor of 90-day mortality and was therefore excluded (p=0.104).

Treatment factors

Resection Margins

The surgical resection margins were classified based on gross and microscopic evaluation. Pathology reports indicated that 32,340 (88.37%) patients had no residual disease, 3,541 (9.68%) had gross residual disease or microscopic tumor cells, and the remaining 717 (1.96%) had indeterminate margins. Patients who had surgical margins positive for residual disease had a 160% increase in odds of mortality within 90 days of surgery (95% CI 2.29-2.94) compared to patients with negative margins, while patients with indeterminate surgical margins experienced no change in odds of 90-day mortality compared to patients with negative surgical margins.

Facility Type

This study analyzed data from patients who were treated at cancer centers with different levels of accreditation by the FACS; 16,923 (46.24%) patients were treated at academic or research centers, 13,007 (35.54%) patients received treatment at community cancer programs, and the remaining 6,668 (18.22%) patients were treated at integrated network cancer programs. Compared to patients treated at an academic center, patients treated at community cancer programs had 23% higher odds of mortality within 90 days of surgery (95% 1.11-1.37), and patients treated at an integrated network cancer program had a 34% increase in odds of 90-day mortality (95% CI 1.18-1.52).

Time to Treatment

In our multivariate analysis, the mean wait time was 37.1±35.0 days. We found that each 1 day increase in time from the date of diagnosis to the date of surgery resulted in a 0.5% decrease in the odds of mortality within 90 days of surgery (95% CI 0.99-1.00). Importantly, we conducted a secondary multivariable regression after classifying delays to treatment as 4 discrete categories: short wait time (<21 days), moderate wait time (22-41 days), long wait time (42-60 days), and very long wait time (>60 days). In this model, wait time was not a statistically significant predictor of overall survival (p=0.24).

Chemotherapy

In our sample, 14,896 (40.70%) patients received surgical resection alone, 11,368 (31.06%) patients received systemic therapy before resection, 7,272 (19.87%) received systemic therapy after surgical resection, 3,010 (8.22%) received systemic therapy at an unknown time frame relative to surgery, and 52 (0.15%) patients received systemic therapy before and after surgery. Our multivariate analysis demonstrated that any chemotherapy conferred a statistically significant survival benefit. Patients treated with chemotherapy both before and after surgery experienced the largest reduction in 90-day mortality (94% reduction) compared to patients not treated with chemotherapy.

Results from the multivariable logistic regression model treating delay to treatment as a continuous variable can be found in Table 1.

**Table 1:** Regression coefficients and confidence intervals from multivariable regression model ^r^ indicates reference group; ^* ^indicates statistical significance at a 5% significance level

	Count/Mean	Percentage/±SD	Odds Ratio (Confidence Interval)
				Demographic Characteristics			
Sex			
Female ^r^	12,736	34.80%	-
Male	23,862	65.20%	1.34 (1.20-1.47)*
Race			
White^r^	28,114	78.82%	-
Black	5,237	14.31%	1.13 (0.98-1.30)
Others	3,247	8.87%	0.67 (0.55-0.82)*
Area			
Large Metropolitan ^r^	20,674	56.49%	-
Small Metropolitan	10,769	29.43%	1.14 (1.02-1.27)*
Non-Metropolitan	5,155	14.09%	1.28 (1.12-1.45)*
Age			
Age	65.9	±11.10	1.04 (1.03-1.05)*
Comorbidities and Social Determinants			
Charlson-Deyo Comorbidity Index Score			
Score = 0 ^r^	24,398	66.66%	-
Score = 1	8,510	23.25%	1.15 (1.04-1.29)*
Score = 2	2,517	6.88%	1.32 (1.12-1.54)*
Score ≥ 3	1,173	3.21%	1.57 (1.26-1.94)*
Insurance			
Medicare ^r^	18,869	51.56%	-
Medicaid	2,354	6.43%	0.90 (0.71-1.15)
Private Insurance	13,932	38.07%	0.86 (0.76-0.98)*
Other	1,443	3.94%	1.06 (0.81-1.39)
Tumor Characteristics			
Histology			
Intestinal Type Adenocarcinoma r	26,125	57.59%	-
				Gastric Stromal	5,816	12.82%	0.24 (0.18-0.31)*
Signet-Ring Cell	6,696	14.82%	1.09 (0.95-1.25)
Others	6,723	14.78%	0.95 (0.83-1.09)
Tumor Grade			
Well-differentiated ^r^	4,097	11.19%	-
Moderately-differentiated	9,949	27.18%	1.43 (1.16-1.76)*
Poorly-undifferentiated	17,941	49.02%	1.80 (1.47-2.21)*
Other Tumor Grades	4,611	12.60%	1.32 (1.03-1.69)*
Clinical Stage			
Stage I ^r^	15,444	42.20%	-
Stage II	11,614	31.73%	1.66 (1.47-1.87)*
Stage III	9,540	26.70%	2.07 (1.82-2.36)*
Treatment Factors			
Tumor Resection Margins			
No Residual Disease ^r^	32,340	88.37%	-
Residual Disease Present	3,541	9.68%	2.60 (2.29-2.94)*
Indeterminate Margins	717	1.96%	1.31 (0.95-1.80)
Treatment Facility Type			
Academic or Research	16,932	46.24%	-
Center ^r^			
Community Cancer Center	13,007	35.54%	1.23 (1.11-1.37)*
Integrated Network Cancer Center	6,688	18.22%	1.34 (1.18-1.52)*
Chemotherapy			
No chemotherapy ^r^	14,896	40.70%	-
Chemotherapy before surgery	11,368	31.06%	0.54 (0.48-0.61)*
Chemotherapy after surgery	7,272	19.87%	0.09 (0.07-0.11)*
Chemotherapy before and after surgery	52	0.15%	0.06 (0.04-0.09)*
Chemotherapy at an unknown time interval	3,010	8.22%	0.64 (0.07-1.22)
Time from Diagnosis to Treatment (continuous)			
Time from Diagnosis to Treatment (continuous)	37.1	±35.06	0.99 (0.99-1.00)*

## Discussion

To evaluate the impact of delays to treatment on patient outcomes, we analyzed the effect of time between diagnosis and treatment on 90-day mortality following gastric cancer surgery in patients with stage I-III gastric cancer using multivariable logistic regression. This model showed that delays to treatment paradoxically resulted in statistically significant improvements in patient survival, calculating that each additional day wait time corresponded to a 0.5% reduction in 90-day mortality rate. Our multivariate analysis also showed statistically significant increases in 90-day mortality after surgery in gastric cancer patients who are older, male, living in a region with fewer than 1 million people, had any medical comorbidities, had worse than well-differentiated tumor histology, had more severe cancer on clinical staging, had residual disease following resection, and were not treated at an academic cancer program. We also observed statistically significant improvements in 90-day survival in patients who do not identify as white or black, patients with private health insurance, and patients who were treated with adjuvant chemotherapy after surgical resection.

Similar to our analysis, a 2019 study by Furukawa et al. also assessed the impact of delays to treatment on overall survival in 696 Stage II/III gastric cancer patients receiving surgery­­­. Patients were divided into the following three groups: short (≤ 30 days), intermediate (> 30 and ≤ 60 days), and long (> 60 and ≤ 90 days) wait time groups. While this study did not identify a relationship between wait time and survival, this study made note of two important considerations. First, survival was significantly worse in the short wait group, and second, the short wait time group also had a statistically higher proportion of patients with worse clinical stage and residual tumor post resection. While the authors mentioned these in their discussion, the study itself did not factor in these potential confounding variables [9]. To address these points in our manuscript, we undertook several steps to validate our results to clarify these aspects.

As Furukawa et al. highlighted, we also noted that patients with shorter wait times tended to have worse overall survival and more severe clinical stage in our sample. A statistically higher proportion of patients with stage III disease (7.2%) experienced 90-day mortality than patients with stage II (6.4%) or stage I (4.5%) gastric cancer (p<0.0001). Moreover, patients with more severe clinical stage had a shorter average wait time as stage III, II, and I patients were operated upon 32.3, 37.3 and 40.0 days after diagnosis respectively (p<0.0001). To evaluate the impact of this relationship on our model, we conducted a subsequent regression analysis using an interaction term to account for the patients with worse clinical stage having shorter wait times. However, in this new model, the interaction term was not a statistically significant predictor of 90-day mortality. Additionally, we conducted an independent regression analysis after classifying wait time using the same discrete categories as Furukawa et al. Similar to their results, our analysis did not show that long wait time (≥30 days as defined by Furukawa et al.) was a statistically significant predictor of overall survival.

Several studies have endeavored to isolate the effect of treatment delays on survival outcomes for gastric cancer patients. A UK study analyzed 115 patients with esophageal or gastric cancer to identify any relationship between delays in diagnosis and operative success rates, finding that no association between the two variables existed for gastric cancer patients [10]. A regression analysis by Kumazu et al. also found no relationship between wait time and survival in 801 gastric cancer patients who underwent curative surgery. Importantly, patients were excluded if they received neoadjuvant chemotherapy [11]. Another study from Japan analyzed the effect of wait times of up to 180 days for gastrectomy in 556 patients with stage I gastric cancer. Patients were stratified into three groups based on wait time: short (<61 days), intermediate- (61-90 days), and long-wait (91-180 days) groups. Overall survival was comparable across the three groups, and multivariable analysis revealed that wait time was not an independent prognostic factor for overall survival [12]. Brenkman et al. published a large, multivariate, multi-site retrospective study in the Netherlands and found no association between wait time, which was treated as a continuous variable, and overall survival in patients treated with gastrectomy alone (n=2077) and patients treated with gastrectomy and neoadjuvant chemotherapy (n=1701) [13]. Kim et al. recently published a retrospective review of Korean patients and demonstrated gastric cancer patients who waited longer than 30 days for treatment (n=766) did not experience increased five-year mortality compared to those who waited less than 30 days (n=1,893) [14]. In sum, the existing literature demonstrates that longer wait time does not adversely impact survival outcomes in gastric. Yet, few studies include stages I-III patients, assess waiting time using both discrete and continuous classifications, address the correlation between wait time and disease severity, and analyze a large, robust sample of patients.

While it is certainly possible that the large sample size in this study allowed for a more granular analysis of the relationship between wait time and patient outcomes, this finding is unlikely to be of any clinical significance. In practice, our findings certainly demonstrate that the time between diagnosis and treatment does not bear negative consequences on patient mortality. Additionally, our results regarding the impact of clinical stage, cancer histology, treatment modality, and the presence of malignant cells postoperatively were consistent with results that have been validated in the literature [15-17]. These findings in a substantial sample of patients are encouraging and can help alleviate patient concerns regarding the potential effect of longer intervals between diagnosis and eventual surgical intervention. Regarding the directionality of our findings, we do not assess that longer wait times would practicably reduce mortality risk. However, we provide convincing evidence that longer wait time does not have a negative impact on mortality for gastric cancer patients.

Our approach to modeling the effect of wait times on 90-day mortality rates following gastrectomy may suffer from inherent limitations. Firstly, the NCDB exists as a collaborative effort from several institutions across the United States. Potentially, this database may include errant inputs or mis-categorizations as a result. Additionally, we are unable to completely control for the effect of heterogeneity in surgical technique on mortality outcomes. However, we believe that significant sample size extracted utilized for our analysis significantly reduces the impact of such confounders. Additionally, our model may suffer from omitted variable bias. However, to account for this possibility, we conducted a thorough literature review of manuscripts studying mortality rates following gastric cancer resection to ensure that our variable list was a superset of variables utilized in the literature. Moreover, to validate the results of previous analyses on the subject, we conducted an additional multivariable logistic regression analysis with delay to treatment as a categorical variable. We found that delay to treatment was not a statistically significant predictor of 90-day mortality following gastric cancer resection. We also conducted a further analysis of the relationship between clinical stage and wait time by attempting to include an interaction variable. We found no statistically significant correlation between clinical stage and 90-day mortality rates following gastrectomy.

## Conclusions

In the majority of existing studies that explore the relationship between waiting time and mortality, the mean or median delay to treatment for gastric cancer patients is within 1-2 months. In such situations, it would be useful to provide patients with sufficient evidence that the delay between diagnosis and treatment will not negatively impact their care. To this end, we assessed the impact of wait time on mortality in a large sample of patients with stages I-III gastric cancer and observed no increase in 90-day mortality with longer wait times. This analysis represents one of the largest datasets in the literature, and assesses the relationship between wait time and mortality in gastric cancer patients using a robust multivariable regression analysis of 36,598 patients. As screening and diagnostic methodologies for gastric cancer become more effective at identifying surgical candidates, decisions concerning when to operate on each patient will have to be made in the context of the prognostic outlook. As such, we feel that this study presents a valuable contribution to the literature by showing that for stage I-III gastric cancer patients undergoing surgery, increased wait time does not adversely impact 90-day mortality.
